# How much do we know about the function of mammalian genes?

**DOI:** 10.1186/s12915-023-01794-w

**Published:** 2023-12-29

**Authors:** Lydia Teboul, Yann Hérault, Sara Wells, Guillaume Pavlovic

**Affiliations:** 1 The Mary Lyon Centre at MRC Harwell, Harwell Campus, Didcot, OX11 0RD Oxon UK; 2 PHENOMIN-Institut Clinique de La Souris, CELPHEDIA, CNRS, INSERM, Université de Strasbourg, Illkirch-Grafenstaden, 67404 Strasbourg, France; 3 Université de Strasbourg, CNRS, INSERM, Institut de Génétique Et de Biologie Moléculaire Et Cellulaire (IGBMC), 1 Rue Laurent Fries, 67404 Illkirch Graffenstaden, France

## Abstract

**The last two decades have seen impressive advances in functional genomics, but we are still a long way** **from understanding the complexity of gene function. Here, we** **pose questions on how much is currently known about the function of mammalian genes and the strategies currently in use to address this issue, and we aim to promote discussion of the challenges that ensue.**

## A work in progress in mammalian genome sequence assembly

The first step required in understanding gene function is to identify the genes themselves, thereby leading to the genome sequence. It took more than twenty years to go from the first draft of the human genome sequence to a Telomere-to-Telomere version [1]. However, from 2000, the initial drafts of mammalian genome sequences served as essential resources to decipher, on a larger scale, the function of mammalian genes. This was carried out through the generation of collections of gene-targeted ES cells and mice [2, 3], supporting the research community in their functional studies and in mining human phenotyping/clinical information [4].

Whilst a reference sequence is an essential cornerstone in understanding gene function on an organismal level, much of the information required to assemble the human pangenome remains missing. The pangenome sequence information will capture the genomic variability between human ethnicities and indeed between individuals in these groups. For example, the NIH “All of us” and “UK Biobank” programmes aim to contribute to filling this gap but much broader population coverage than the one currently available will be required for a complete human pangenome sequence [5].

In parallel, pangenome sequences are being assembled for animal models, but fundamental information such as accurate numbers of coding genes has not yet been fully determined [6] and genomic diversity of laboratory strains does not necessarily mirror that of the human genome, both in extent of variation (amount of diversity) and extent of homologous variation.

Thus, while complete drafts of genome sequences exist for many species, we are still at the early ages of assembling sequences of mammalian genomes that provide an integrated understanding of gene function, and many challenges remain, both in sequence completion itself, and in capturing genetic diversity and its role.

## What do we know of gene function?

The function of mammalian genes has been much studied, and a large body of information is already available, with active research on individual genes in traditional laboratory research settings, and large-scale functional annotation programmes that employ cell culture models and animals all contributing for an initial description of the function of the majority of coding mammalian genes. This has resulted in a rich functional gene annotation for increasing numbers of species [7, 8].

However, this initial body of knowledge has begun to also reveal that understanding gene function is a much more complex endeavour that goes beyond the simple interrogation of genes for a given biological function, one at a time. The complexity stems from a combination of factors such as the potential diversity of gene products generated from a given gene through mechanisms such as alternative splicing and editing, the pleiotropic aspect of gene function, the diversity of alleles within and between populations, and the combinatorial dimension of gene–gene interactions.

In addition, comparatively little is currently known of the activity of other genetic functional units such as non-coding genes, regulatory sequences, or the 3-dimensional organisation of the genome [2]. It is also noteworthy that genetic studies of laboratory models most often, and pragmatically, focus on aspects that are relevant to human biology.

## What research models do we have to understand the function of mammalian genes?

Biomedical research has generated a vast diversity of models, both in *vitro* and in *vivo*. These include many non-mammalian models (for example, yeast, drosophila and zebrafish) that have provided much insight about the function of mammalian protein-coding genes.

The breadth of models, in health and disease states, spans cultured cell lines that reflect the diversity of cell types in organisms, as well as laboratory animal species, with a few of the latter having been particularly well-studied (drosophila, mouse, zebrafish and rat). With research efforts that span whole genomes having been conducted over the past two decades in many experimental models (for example [2, 9]), the integration of all publicly available phenotyping information (from large-scale consortia and individual research laboratories) into curated databases now facilitates the understanding of gene function across multiple body systems and species [5]. Genetic studies take advantage either of natural variation or of engineered mutations that alter or ablate the function of genes as a result of deletions or more discrete sequence changes. Structural variation—the rearrangement of large stretches of DNA—also has profound implications in understanding evolution and human disease and is increasingly being studied. Thanks to genome editing, models of structural variation have also become much easier to obtain in recent years.

At the same time, the diversity and sophistication of in vitro models (examples of which include organoids, assembloids and multi-organ culture systems) has dramatically increased, bringing onto the horizon vast potential for enhancing and replacing animal models. In addition, genome-editing technologies have greatly enhanced our capability for genetic manipulation in all model systems, transforming functional genomics by accessing both new species and the diversity of genetic backgrounds within species.

## Are our models fit for the new challenges?

The scope of the work carried out to date highlights the limitations of current models in capturing the vast complexity of gene function(s) within a living organism. For instance, both financial constraints and the appeal of building on information from a previous study mean that researchers have a tendency to return to the same models for genetic studies. Furthermore, an immediately evident limitation of the state of the art is that most models address gene function(s) by mutating one gene at a time, and often only focus on specific aspects of biology for which the model was generated to interrogate, neglecting the pervasive more pleiotropic aspects of gene function. Additionally, much of the focus has been on protein-coding genes, leaving the function of other types of DNA elements much less understood. This is compounded by the fact that most functional studies are performed in the laboratory mouse, using a small number of standard inbred genetic backgrounds, while human genetic research has primarily focused on male Caucasian individuals. These issues are starting to be recognised and addressed by extending coverage to a more diverse population [10].

Whilst sophisticated in vitro models continue to progress, they have not yet been able to replace animal models to fully recapitulate the biology of complex systems or maintain cellular integrity comparable to an in vivo state. And despite the complexity of current in vivo modelling, much development is also required to address the complex questions of host–environment relationship. Equally, standard phenotyping tests used to characterise many of the available laboratory animal models bring a host of biases that hinder the study of gene function.

## What do we require from the next generation of models?

After sequencing the genomes and interrogating the function of protein-coding genes, a different type of genomic study is increasingly required. The questions asked are more complex to address, requiring both new models and new phenotyping paradigms.

Ideally, this will extend our understanding to all genetic elements—whether coding, non-coding, structural or epigenetic—and the full extent of their function(s). This would mean accessing models that will address all functional elements and studying them at various levels (molecular, cellular, organ, organism and population) for all aspects of biology. Practically, this means extending our range of biological models to address the function of other genetic elements with at least the same depth as that of the coding genome. This has become much more feasible with our recent ability to engineer genomes at will. Notably, for non-coding elements, orthology is much less evident, which brings new challenges to model design and systematic mutagenesis approaches.

An additional dimension of complexity exists in that, for each genetic element, we also need to understand the impact made by different allelic variants within its network and in its genomic context, which is an essential aspect of biomedical research. This requires extending the use of polygenic models, ensuring variation of genetic backgrounds and including population studies. The molecular toolbox for genetics (for example, reporter or conditional alleles) that was previously limited to a few animal species/backgrounds can now be applied to these complex models. These highly sophisticated paradigms will be essential to capture the next layer of biological complexity.

Finally, gene function is evidently modulated by ageing and the environment—all the extrinsic factors that modulate the expressivity of gene function—of the organism. This ultimate level of complexity can take many forms, including, for example, physical conditions (such as temperature and light), the presence of pathogens or the availability of nutrients. This results in a potentially infinite number of combinations of genetic and environmental variables, which provide unique challenges for functional genetics studies.

To answer these complex questions will require many additional tools beyond modern genetic models. These will include phenotyping paradigms that can produce increasingly sophisticated datasets (for example, combined omics and live imaging), as well as mathematical and computational approaches with which to analyse these data.

The more we understand about the genome, the more we can appreciate just how much the function of genes is an extraordinarily complex question and how little we know about it. Much has been learned since the initial drafts of mammalian genome sequences became available, but much, in terms of better models and methods of analysis, is still required to advance our understanding of functional genomics and effectively move towards personalised medicine.

## Data Availability

Not applicable.
